# Clarifying Water

**Published:** 1879-01

**Authors:** 


### CLARIFYING WATER.

Thirty years ago, in travelling up and down the Missisippi river whose waters, below the mouth of the Missouri, were so turbid that it was impossible to see through a glass, it was a common amusement to tie a bit of alum to a thread and letting it down into the water give it a swinging motion for a minute, and in a few minutes more, the water would be as clear as a rain drop. Within a short time the statement has appeared in the public prints as if it were something new. It may however be well to add the exact proportions in which the alum should be used so as not to make the water taste of it. Three quarters of a pound of finely pulverized alum stirred well into a ton of water; in smaller quantities to each quart of water, four grains of Alum. The Sulphate of Alumina is greatly better than the Rock or Potash Alum, as it introduces no alkaline matter into the water. This shows how easy it may be for practical items of knowledge to drop out of sight, at least for a time, and that too with all the advantages of the printing press; hence it is no wonder that valuable arts have been lost to the world before the discovery of types, such as embalming, certain works in glass, etc.

